# Micromotion-based balanced drilling technology to increase near cortical strain

**DOI:** 10.1186/s12893-022-01816-4

**Published:** 2022-11-11

**Authors:** Yang Wang, Qiang Zou, Zhanchao Wang, Wei Wang, Hao Shen, Hua Lu

**Affiliations:** 1 Department of Orthopaedics, Xinhua Hospital Affiliated to Shanghai Jiaotong University School of Medicine, Chongming Branch, 202150 Shanghai, China; 2 Department of Orthopaedics, Xinhua Hospital Affiliated to Shanghai Jiaotong University School of Medicine, No. 1665 Kongjiang RD, Shanghai, 200092 China

**Keywords:** Internal fracture fixation, Femoral fractures, Femur

## Abstract

**Objective:**

A micromotion-based balanced drilling system was designed based on a locking plate (LP) and far cortical locking (FCL) concept to maintain the balance of micromotions of the cortex on both sides of a fracture region. The system was tested by axial compression test.

**Methods:**

The fracture gap was set to 2 cm, and locking screws with a diameter of 5 mm and a locking plate were used to fix it. The diameters of the two sections of the stepping drill were 3.5 mm and 5.0 mm, respectively. One of the matching drilling sleeves was a standard sleeve (eccentricity, 0 mm) and the other was an eccentric sleeve (proximal eccentricity, 1 mm). A model of the fixed locking plate (AO/ASIF 33-A3) for distal femoral fractures with a gap of 2 cm was established based on data from 42 artificial femurs (SAWBONE). According to the shape of the screw holes on the cortex, the fixed fracture models were divided into a control group (standard screw hole group X126, six cases) and an experimental group (elliptical screw hole group N, 36 cases). The experimental group was further divided into six subgroups with six cases in each (N126, N136, N1256, N1356, N12356, N123456), based on the number and distribution of the screws on the proximal fracture segment. The control, N126, and N136 groups were subjected to an axial load of 500 N, and the other groups were subjected to an axial load of 1000 N. The displacements of the kinetic head, far cortex, and near cortex were measured. The integral structural stiffness of the model and the near cortical strain were calculated. The data of each group were analyzed by using a paired *t*-test.

**Results:**

When the far cortical strains were 2%, 5%, and 10%, the near cortical strains in group N126 were 0.96%, 2.35%, and 4.62%, respectively, significantly higher than those in the control group (X126) (p < 0.05). For a different distribution of the screws, when the far cortical strains were 2%, 5%, and 10%, the near cortical strains in group N126 were significantly higher than those in group N136 (p < 0.05). However, there was no significant difference between the near cortical strains in the two groups with four screws (p > 0.05). For different numbers of screws, the near cortical strains in the three-screw groups were significantly higher than those in the four-screw groups (p < 0.05), and there was no significant difference in near cortical strains among the four-, five-, and six-screw groups (p > 0.05).

**Conclusion:**

The proposed drill and matching sleeves enabled a conventional locking compression plate to be transformed into an internal fixation system to improve the balanced motion of the near and far cortices. Thus, strain on a fracture site could be controlled by adjusting the diameter of the drill and the eccentricity of the sleeve.

## Introduction

Distal femoral fractures account for 3%–6% of adult femoral fractures and 0.4% of all fractures, and have extremely high rates of disability and mortality [1, 2]. Open reduction and internal fixation surgery are currently recognized treatments for distal femoral fractures [3]. Internal fixation provides the best mechanical stability and accelerates the healing of the fracture, allowing for early movement of the injured limb [4]. The strain on the fracture site is key to its healing, where an excessive axial stress and shear force are not conducive to healing. The magnitude of the strain is related to the gap of the fracture and the relative displacement between the fractured pieces, which largely depends on the stiffness of the fixed structure. Regarding the importance of strain in fracture healing, Perren claimed that the strain at the fracture end must be controlled to between 2 and 10% during secondary healing [5, 6]. Simple fractures require anatomical reduction. A strong fixation can reduce the strain at the fracture end, and the fracture can heal through primary healing [4]. Comminuted fractures require a more elastic structure to increase the micromotion between fracture blocks, keep the ends of the fracture relatively stable, promote the formation of calluses, and achieve secondary healing [7–9]. Comminuted fractures can tolerate more interfragmentary motion than simple fractures because the entire motion in them is shared by multiple, smaller fracture spaces [10]. However, excessive interfragmentary motion may lead to the hypertrophy-induced nonunion of a fracture, and too little interfragmentary motion may cause bone atrophy [11]. Although the ideal stiffness and motion cannot be determined, overall structural stiffness can be modified by choosing different implants, screw types, and positions of the screw or plate [12, 13]. When the stability is too high (in other words, the strain is too low), it may result in nonunion [14].

A locking plate is the most commonly used implant used to manage distal femoral fractures because it increases overall stiffness by stabilizing the angle of the screw [15]. Although the clinical efficacy of a locking plate is promising, such attendant complications as delayed healing, implant failure, and non-union are common [4].

In response to the high stiffness and the formation of small or uneven calluses due to the fixation of the locking plate, Bottlang and Feist proposed the use of far cortical locking (FCL) technology [16]. FCL allows for limited axial movement of the cortex (near cortex) beneath the plate to promote the symmetrical formation of the callus at the near and far cortices. At present, only one company provides a system that uses FCL technology in high expense. Analyses of its design and mechanical properties have revealed that although the axial activity of near cortex increases; it is still limited and insufficient compared with that of the far cortex [17]. In addition, the hole of the near cortex is round, which can cause additional rotational shear and lateral movement that can impair the formation of the callus between the ends of the fracture.

The study seeks a solution that can increase the axial movement of the bone cortex on the side of the plate to compensate for flaws in the FCL design, promote uniform displacement between fracture blocks, and induce symmetrical callus formation with a lower burden on the patient. A micromotion-based balanced drilling system was designed by using the current conventional locking plate and the far cortical locking concept. This required changes to only its drill and sleeves. The diameter of each section of the stepping drill depended on the diameter of the screw to be used. The eccentricity of the sleeve depended on the gap between the ends of the fracture. The diameter of the far cortex hole was the same as that of the locking screw rod to ensure that the locking screws and the far cortex were interlocked. The holes of the screw of the near cortex were oval. Their long diameter increased as the diameter of the screw and the fracture gap. This may provide a condition for balanced micromotions of the cortex on both sides of the fracture site. The other purpose of this study was to subject the proposed technique to mechanical tests to determine whether it could allow for the uniform displacement of the near and far cortices under normal physiological load, and control the strain at the ends of the fracture to between 2 and 10%.

## Methods

### Experimental materials and equipment

**Fig. 1:**
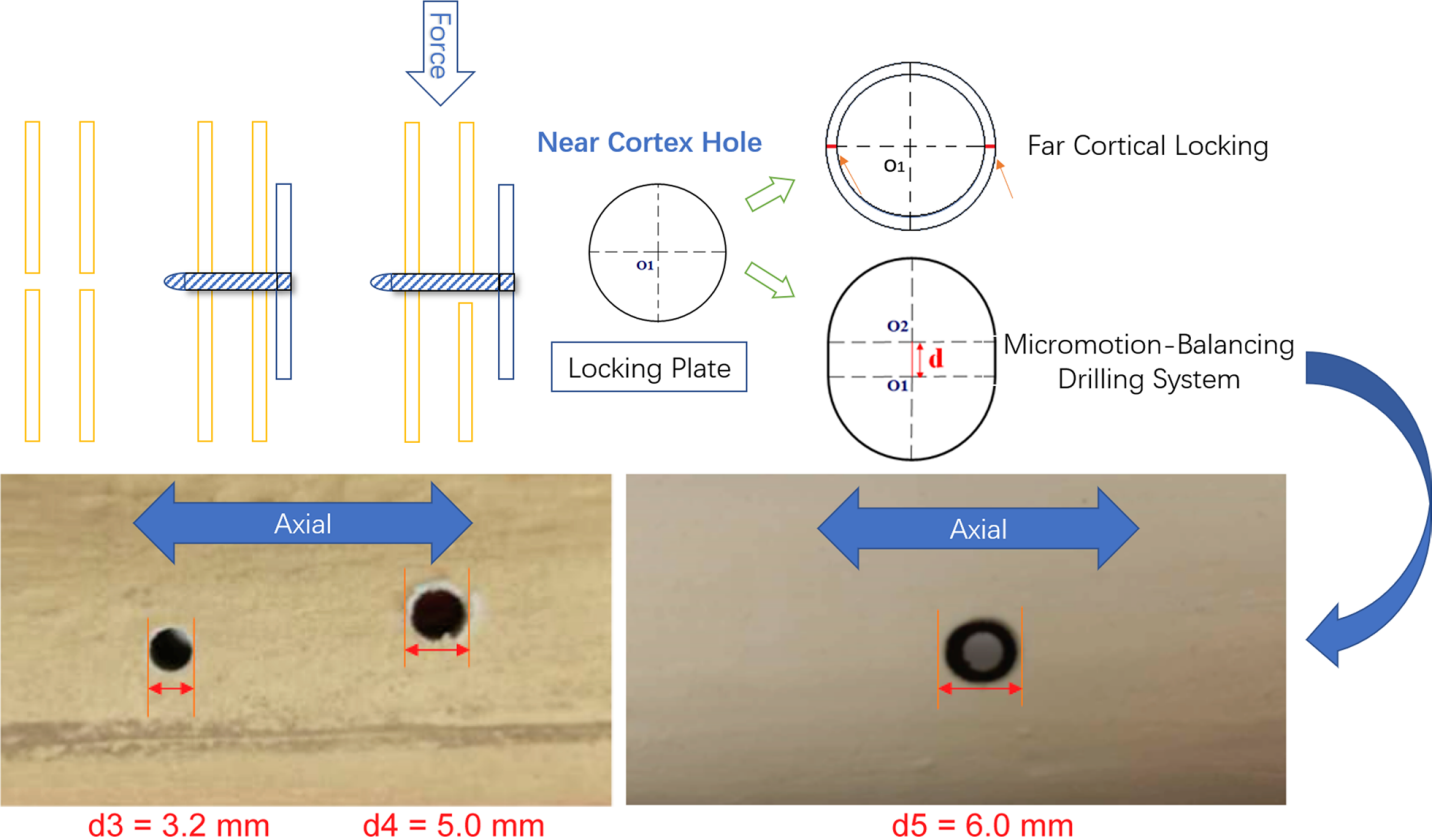
Comparison of the diameters of holes between the standard method and the proposed drilling method. The d3 was created by a standard 3.2 mm drill, d4 was created by the proposed drill with a standard sleeve, and d5 was created by the proposed drill and two sleeves

**Fig. 2:**
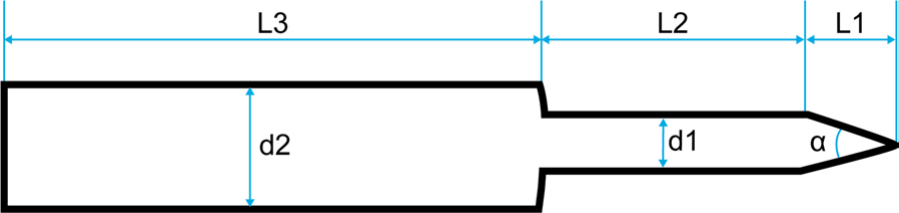
Design model of the drill

**Fig. 3:**
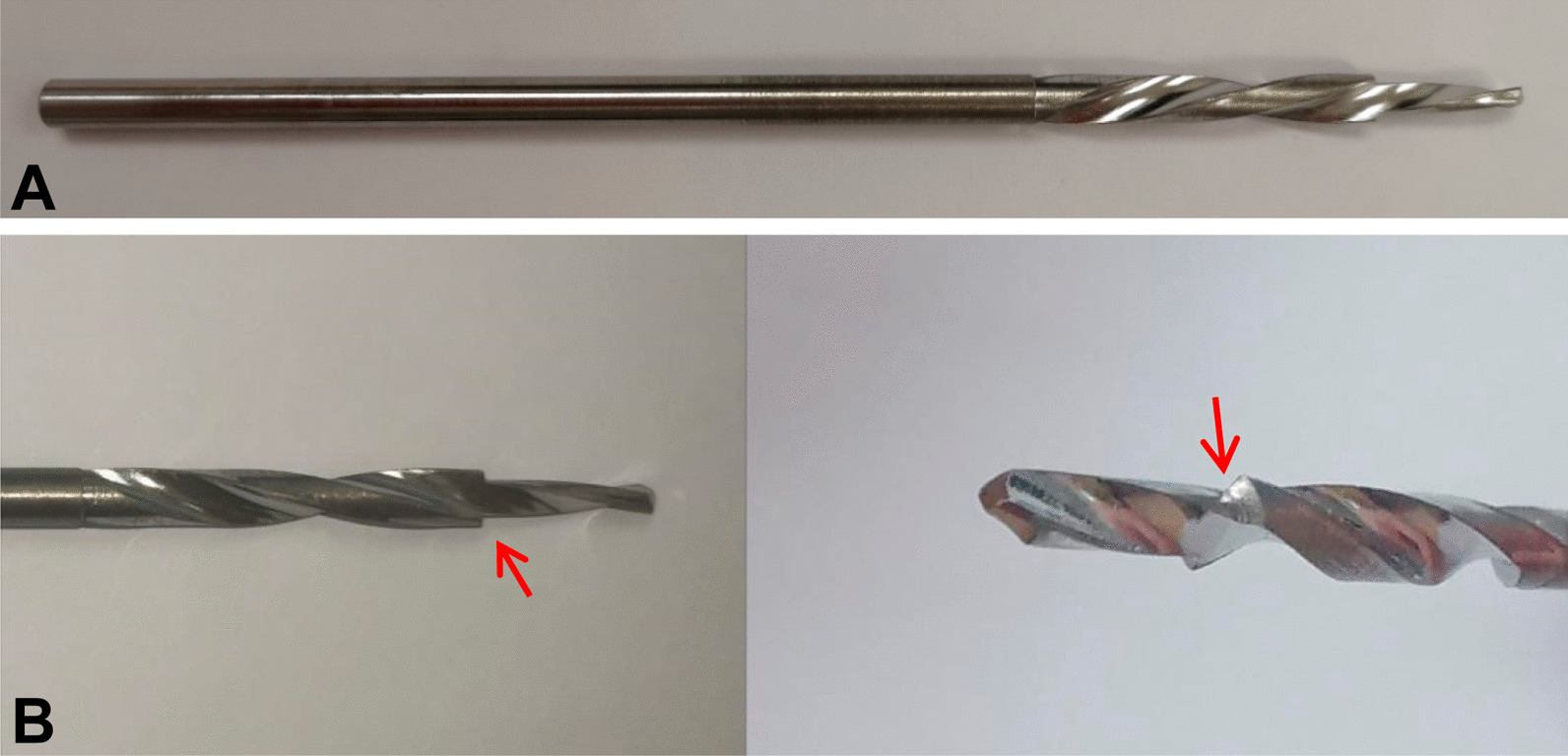
**a** The entire drill. **b** Stepping design of the drill. The steps are indicated with arrows

Stepping drill: In this study, the fracture gap was set to 2.0 cm (comminuted fracture). In order to achieve secondary healing, the far and near cortical strains needed to be controlled to between 2 and 10%. A strain of 5% (1 mm, half of 10%) was set as the target value. The length of the near cortical oval screw hole was determined to be 6 mm (1 mm plus a diameter of the screw thread of 5 mm), and its direction was parallel to the long axis of the femur (Fig. 1). The diameter and length of each section of the drill are shown in Fig. 2: d1 is the diameter of the standard drill, 3.2 mm, d2 is the diameter of the screw thread, 5.0 mm, L1 was 2 mm, L2 was 15 mm, and L3 was 100 mm. Figure 3a shows the drill and Fig. 3b shows its stepping design. The stepping drill was just penetrated through the far cortex. The diameter of the conventional hole is 3.2 mm, while the micromotion-based balanced drilling system could make a oval screw hole with a long diameter of 6 mm and a short diameter of 5 mm. The drilling speed was decided by electric drill(Rotate speed 1200r/min, Torque 4 N·M).

**Fig. 4:**
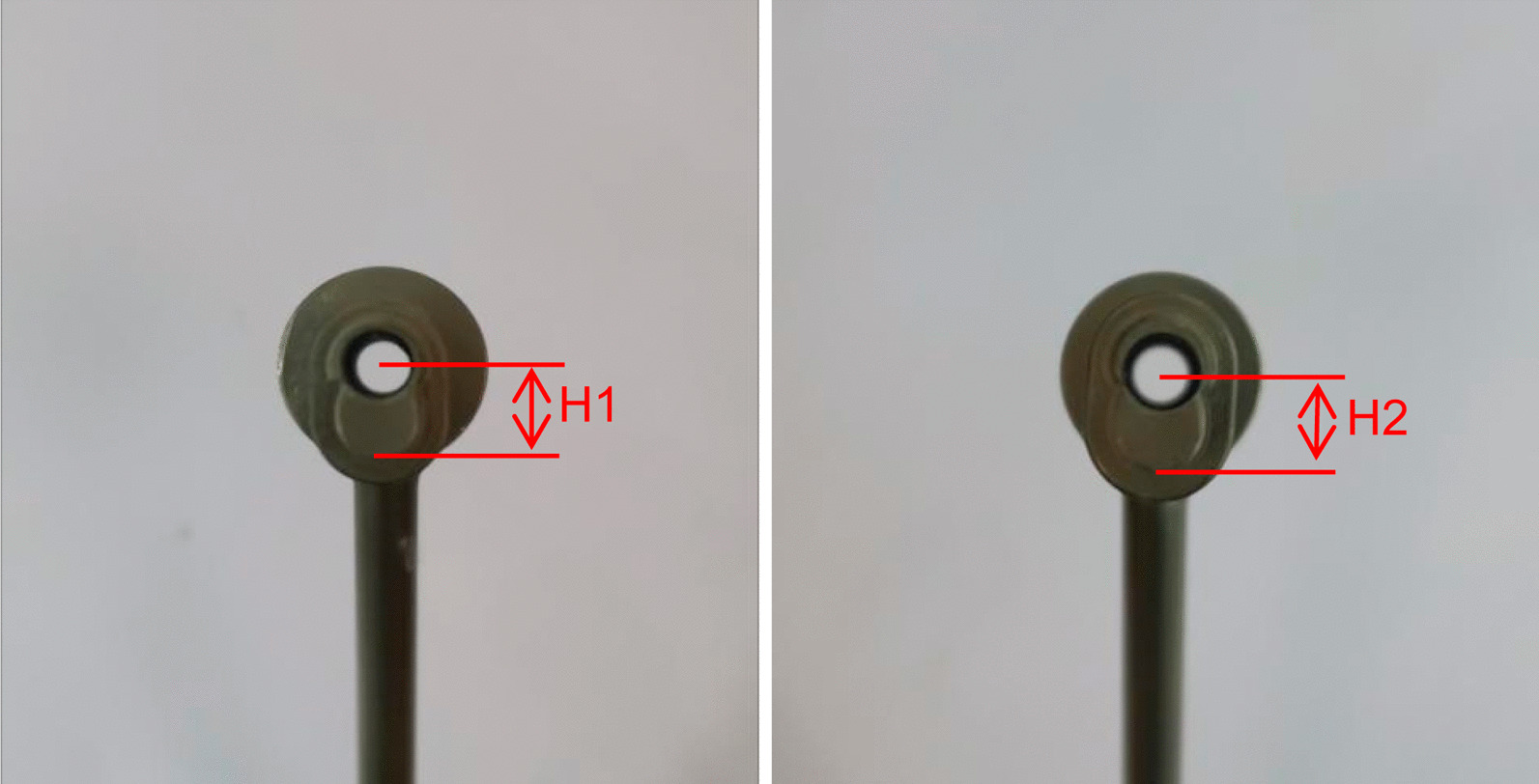
Anterior view of a standard sleeve with an eccentricity of 0 mm and an eccentric sleeve with a proximal eccentricity of 1 mm. H1 is the distance between the proximal end of the standard sleeve and the center of the drilling hole. H2 is the distance between the proximal end of the eccentric sleeve and the center of drilling hole

**Fig. 5:**
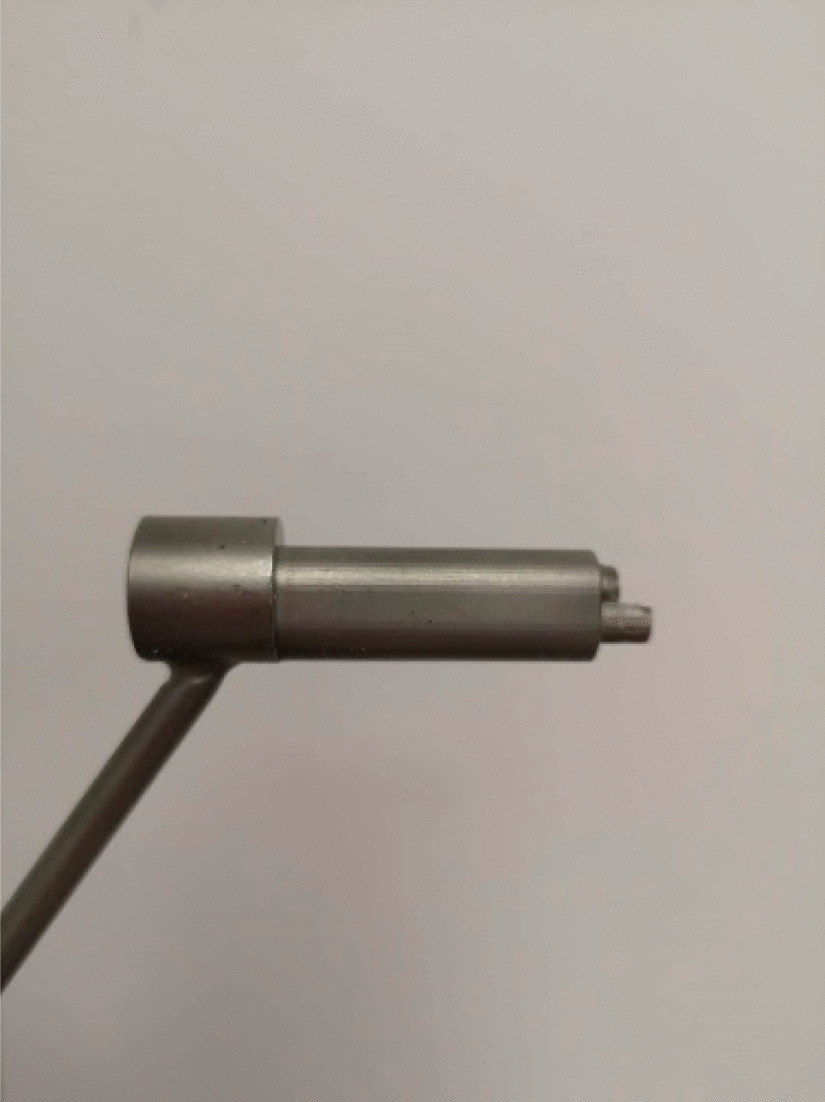
Lateral view of the sleeve

- Matching sleeves: The two sleeves (Fig. 4) used were a set of standard sleeves, with centers identical to the center of the hole on the locking plate. The near eccentric sleeve had an eccentricity of 1 mm (Fig. 4 shows the 1 mm difference between H1 and H2). Figure 5 shows the side view of the sleeve. The drilling effects of the drill and matching sleeves are shown in Fig. 1.
- Forty-two left artificial femurs (SAWBONE, Shanghai Yino Culture Communication Co., LTD Company, FEL201912): Forty-two, six-hole left lateral femoral anatomical locking plates (titanium alloy, combined hole), 66 titanium alloy screws with a diameter of 5.0 mm and length of 40 mm, and 354 titanium alloy locking screws with a diameter of 5.0 mm and length of 55 mm were used.
- Mechanical test equipment: Instron 5569 mechanical tester (Norwood, MA, USA).
- Data collection: Bluehill 2 (Instron, USA).
- Image acquisition and analysis: VIC-3D (XR-9 M, Correlated Solutions Company, Westford, MA, USA).

### Fracture fixation model and grouping

**Fig. 6:**
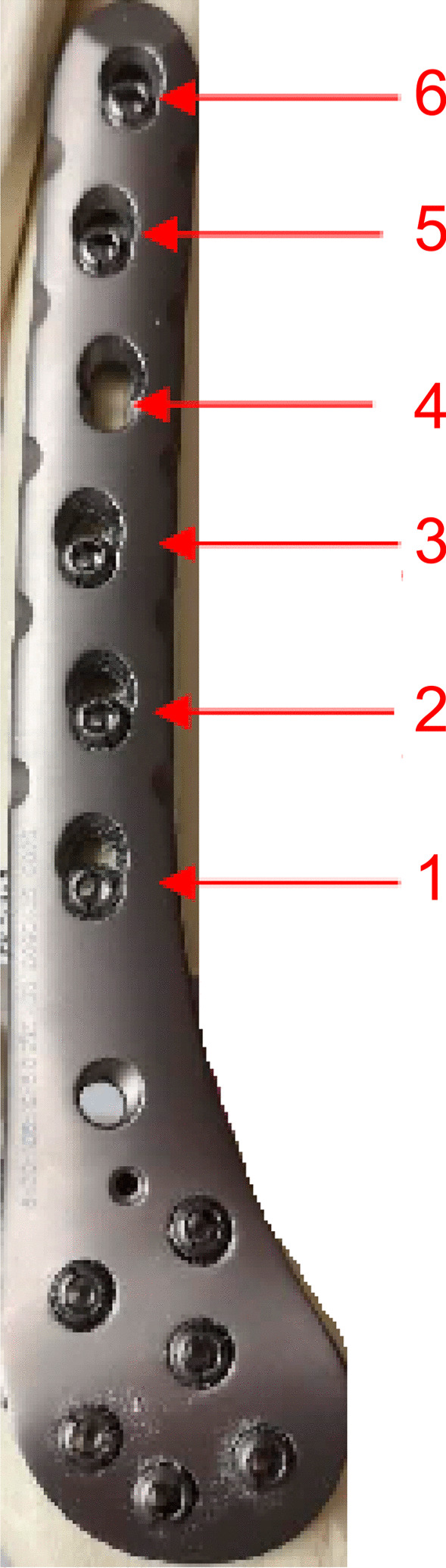
Positions of the six drilling holes on the fixation plate

**Fig. 7:**
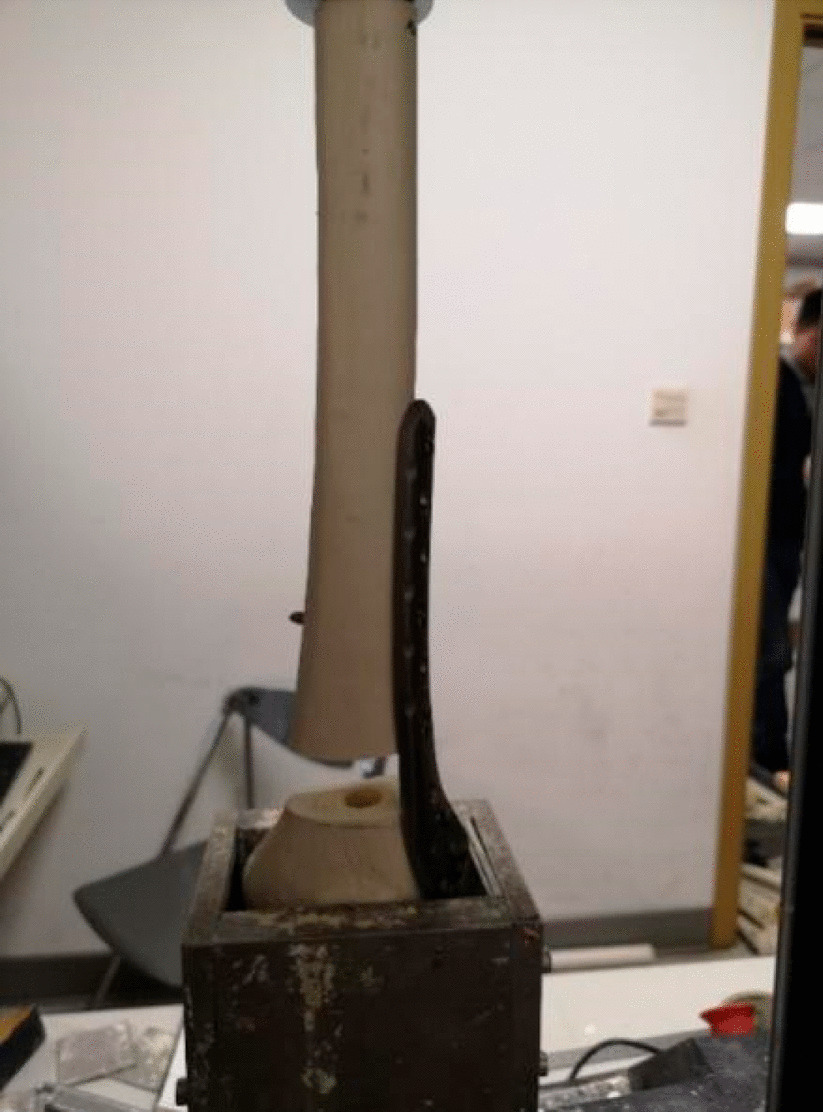
Anterior view of fixation of the distal femoral fracture model

- Fracture model: In the 42 left artificial femurs, a horizontal osteotomy at a distance of 4.5 cm from the distal articular surface and a fracture model with a gap of 2 cm were established to simulate a comminuted fracture (AO/OTA 33-A3) (Fig. 7).
- Grouping: The 42 fracture models were divided into a control group and an experimental group. For the six cases in the control group (i.e., standard screw hole group, group X, X126), we used standard sleeves and drill. A six-hole distal femoral anatomical locking plate was used to fix the fracture. The femoral condyle was drilled with a standard sleeve, and six locking screws with a diameter of 5.0 mm and length of 55 mm were screwed into it. Drilling holes were made at positions 1, 2, and 6 at the proximal end of the fracture (“1” was closest to the fracture line and “6” was the farthest from it). For position 1, a titanium alloy screw with a diameter of 5.0 mm and length of 40 mm was used. For positions 5 and 6, titanium alloy screws with a diameter of 5.0 mm and length of 55 mm were used. The experimental group (i.e., the elliptical screw hole group, group N) was also fixed with the same steel plate. The screws and drilling methods at the part distal to the fracture were the same as those in the control group. For the proximal segment, the newly designed drill and sleeves were used to form an eccentric screw hole (i.e., eccentric to the proximal end). The locking screws were sequentially screwed in, in accordance with the grouping criterion. According to the number and distribution of screws in the part proximal to the fracture, the experimental group was divided into six subgroups (i.e., N126, N136, N1256, N1356, N12356, and N123456) with six cases in each group. The arrangement and grouping of the screws are shown in Table 1 and Figs. 6, 7.

**Table 1:** Arrangement and grouping of screws in the distal femoral fracture model

Position	X126	N126	N136	N1256	N1356	N12356	N123456
1	X	N	N	N	N	N	N
2	X	N		N		N	N
3			N		N	N	N
4							N
5				N	N	N	N
6	X	N	N	N	N	N	N

### Biomechanical test: Axial compression test

**Fig. 8:**
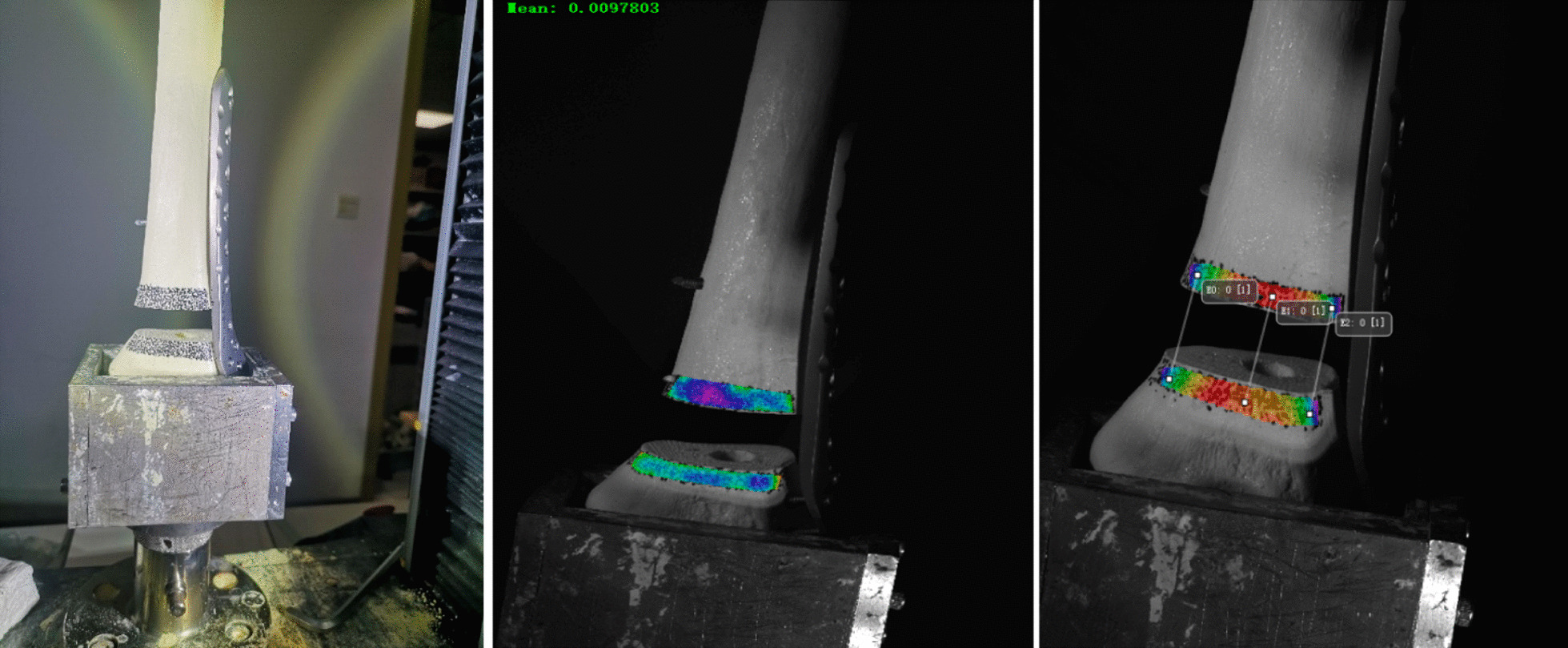
Pictures collected and processed by the VIC

The fracture fixation model was placed in the groove and plaster was poured to fix it. The overall preload was 10 N. The speed of descent of the pressure head of the Instron mechanical tester was 2 mm/min. Axial loads of 500 N were applied to the control, N126, and N136 groups, and 1000 N was applied to the other groups. We used VIC-3D to acquire images (Fig. 8), and calculated the displacement of the near cortex and far cortex of the proximal end of the fracture. A curve relating the force and displacement data collected by the pressure head was drawn and the overall stiffness was calculated. The corresponding values of the displacement of the near cortex when the far cortical strains were 2% (0.4 mm), 5% (1.0 mm), and 10% (2.0 mm) were noted.

### Statistical analysis

Paired *t*-tests on SPSS 18.0 software were performed on the values of the load and near cortical displacement between the groups when the far cortical strains were 2%, 5%, and 10%. For p < 0.05, the difference was considered statistically significant.

## Results

When the far cortical strains were 2%, 5%, and 10%, the corresponding near cortical strains in the group N126 were 0.96%, 2.35%, and 4.62%, respectively, all greater than those in the control group (p < 0.05). The load of group N126 was not different from that of the control group (p > 0.05); however, when the far cortical strain reached 10%, the load of the experimental group was 759.77 ± 201.64 N, which was 1.46 times greater than that of the control group.

In the experimental group, when the f cortical strains were 2%, 5%, and 10%, the proximal cortical strains of the three-screw groups (N126) were 0.91%, 2.35%, and 4.62%, respectively, greater than those of the other subgroups (p < 0.05).

**Fig. 9:**
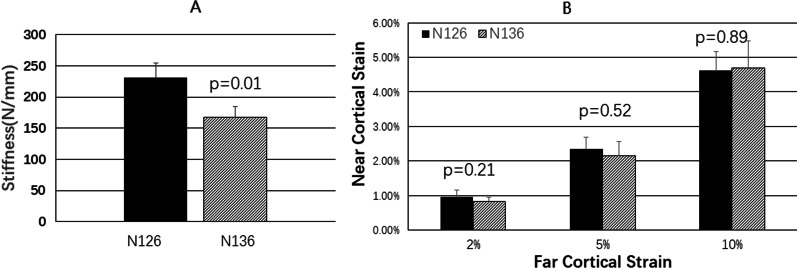
**a** Comparison of structural stiffnesses of groups N126 and N136. **b** Comparison of near cortical strains of groups N126 and N136

**Fig. 10:**
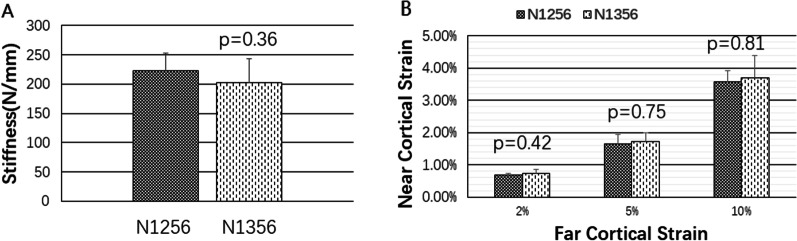
**a** Structural stiffnesses of groups N1256 and N1356. **b** Proximal cortical strains of groups N1256 and N1356

In the three-screw groups, the closer the middle screw was to the fracture line, the greater the structural stiffness was (p < 0.05, Fig. 9a), with no difference in the proximal cortical strain (p > 0.05, Fig. 9b). The structural rigidity and proximal cortical strain of the four-screw groups were not affected by the distribution of the screws (p > 0.05, Fig. 10a and b).

**Fig. 11:**
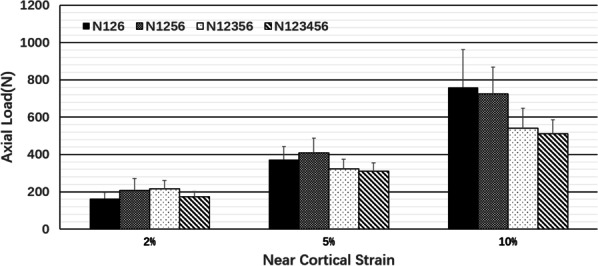
Comparison of axial loads for different numbers and distributions of screws at the proximal segment of the fracture

In the experimental group, when the distal cortical strains were 2% and 5%, there was no difference in the load of each group (p > 0.05); however, when the distal cortical strain was 10%, the loads of the three- and four-screw groups were greater than those of the five- and six-screw groups (p < 0.05, Fig. 11).

## Discussion

### Micromotion-based balanced drilling technique

Inspired by the design concept of the FCL, A micromotion-based balanced drilling technology was designed and tested to further increase the axial displacement of the near cortex under a steel plate. This technology compensated for the shortcoming in the FCL design, promoted axial displacement and inhibited horizontal displacement. This technique still used the locking plate system, but with a newly designed drill and matching sleeves. The drill was designed in a stepping shape. The diameter and length of each segment could be determined according to the diameters of the screw and the femur, and the eccentricity of the sleeve could be determined according to the fracture gap. In this study, screws with a diameter of 5.0 mm were used; hence, the diameter of the L2 segment (d1) of the drill was 3.2 mm and that of the L3 segment (d2) was identical as the diameter of the screw thread (5.0 mm). In this way, the screws engaged only with the far cortex, and could slide in the near cortical hole. The fracture model of this study simulated a segmental comminuted fracture. The fracture gap was set to 2 cm and the expected strain was 5%. Therefore, we set the eccentricity of the sleeve to 1 mm.

Due to the loss of cortical support, comminuted fractures require a relatively stable mechanical environment provided by internal fixation so that the interference caused by bone cortical contact can be ruled out. The results showed that strain at the near cortex in the experimental group was significantly higher than in the control group, and the overall structural stiffness improved at the same time. Thus, the results are similar to the results for axial stiffness in case of FCL under larger loads (i.e., larger than 500 N) reported by Bottlang et al. They claimed that the extra support provided to the near cortex increased structural rigidity sixfold [18]. When the far cortical strains were 2%, 5%, and 10%, the near cortical strains of the experimental group were 0.96%, 2.35%, and 4.62%, respectively. There was still a gap to the corresponding far cortical strain that could not yield complete balance; however, a significant improvement was noted compared with the control group. When the far cortical strain reached 10%, the load of the experimental group reached 759.77 ± 201.64 N. Although there was no statistically significant difference compared with the control group, the load-bearing capacity had a clear tendency to increase (i.e., 1.46 times the original load), thus helping patients to perform weight-bearing exercises early on in their recovery. While premature weight-bearing by a heavy patient (500 N) may lead to the unbalanced micromotion of the near and far cortical callus and, eventually, lead to the failure of internal fixation.

### Different numbers and distributions of screws

The results showed that the strain of the cortex under the plate of the three-screw experimental groups was significantly greater than that of the other experimental groups, indicating that the micromotion balance in this group was better than that in the other groups. This suggested that for this type of fracture, if only the axial load was considered, fixation with three screws at the proximal segment was sufficient. Three screws not only ensured good micromotion balance, but also reduced the operating time and the cost. Groups with three screws at the proximal segment (X126 and N126) were chosen for the greater stiffness and fewer strain that could reduce the systematic error. In clinical practice, such fractures are often fixed with three screws on each side of the fracture gap to achieve good stability [19–22].When comparing the distance in case of the three screws with different arrangements, the results showed that when the middle screw was far from the fracture line, the overall stiffness and the near cortical strain decreased significantly (p < 0.05). These results are similar to those reported by Stoffel et al. [23]. We also observed that the nearest one to two screws on both sides of the fracture gap bore most of the load [24].

## Conclusion

The stepping drill and matching sleeves could transform an ordinary locking plate into an internal fixation system that enabled the distal and proximal cortices to achieve optimal micromotion balance. By adjusting the diameter of the drill and the eccentricity of the sleeve, the strain on the far and near cortices of the fracture could be controlled. This system is suitable for fractures in which the medial and lateral cortices of the distal femoral metaphysis are severely crushed. Compared with FCL and double plates, it was more conducive to the healing of fractures and reducing medical costs.

To increase the predictability of the effect of this technology on the human body, studies involving torsional and bending mechanical tests and experiments on animal are needed in the future.

## Data Availability

The datasets used and/or analyzed during the current study are available from the corresponding author on reasonable request.
