# Correction: Micromotion‑based balanced drilling technology to increase near cortical strain

**DOI:** 10.1186/s12893-022-01856-w

**Published:** 2022-11-23

**Authors:** Yang Wang, Qiang Zhou, Zhanchao Wang, Wei Wang, Hao Shen, Hua Lu

**Affiliations:** 1 Department of Orthopaedics, Xinhua Hospital Affiliated to Shanghai Jiaotong University School of Medicine, Chongming Branch, Shanghai, 202150 China; 2 Department of Orthopaedics, Xinhua Hospital Affiliated to Shanghai Jiaotong University School of Medicine, No. 1665 Kongjiang RD, Shanghai, 200092 China

## Correction: BMC Surgery (2022) 22:387 10.1186/s12893-022-01816-4

Following the publication of the original article [1], the co-first author name 'Qiang Zhou' has been misspelled as 'Qiang Zou'.

The original article has been corrected.
